# Construction of the Six-lncRNA Prognosis Signature as a Novel Biomarker in Esophageal Squamous Cell Carcinoma

**DOI:** 10.3389/fgene.2022.839589

**Published:** 2022-03-31

**Authors:** Ze-Jun Zheng, Yan-Shang Li, Jun-De Zhu, Hai-Ying Zou, Wang-Kai Fang, Yi-Yao Cui, Jian-Jun Xie

**Affiliations:** ^1^ Department of Biochemistry and Molecular Biology, Shantou University Medical College, Shantou, China; ^2^ Department of Pathology, Medical College of Jiaying University, Meizhou, China; ^3^ Department of Thoracic Surgery, Beijing Friendship Hospital, Affiliated to the Capital University of Medical Sciences, Beijing, China

**Keywords:** esophageal squamous cell carcinoma, long non-coding RNAs, prognosis, machine learning, LASSO, LINC01273

## Abstract

Esophageal squamous cell carcinoma (ESCC) is a common malignant gastrointestinal tumor threatening global human health. For patients diagnosed with ESCC, determining the prognosis is a huge challenge. Due to their important role in tumor progression, long non-coding RNAs (lncRNAs) may be putative molecular candidates in the survival prediction of ESCC patients. Here, we obtained three datasets of ESCC lncRNA expression profiles (GSE53624, GSE53622, and GSE53625) from the Gene Expression Omnibus (GEO) database. The method of statistics and machine learning including survival analysis and LASSO regression analysis were applied. We identified a six-lncRNA signature composed of AL445524.1, AC109439.2, LINC01273, AC015922.3, LINC00547, and PSPC1-AS2. Kaplan–Meier and Cox analyses were conducted, and the prognostic ability and predictive independence of the lncRNA signature were found in three ESCC datasets. In the entire set, time-dependent ROC curve analysis showed that the prediction accuracy of the lncRNA signature was remarkably greater than that of TNM stage. ROC and stratified analysis indicated that the combination of six-lncRNA signature with the TNM stage has the highest accuracy in subgrouping ESCC patients. Furthermore, experiments subsequently confirmed that one of the lncRNAs LINC01273 may play an oncogenic role in ESCC. This study suggested the six-lncRNA signature could be a valuable survival predictor for patients with ESCC and have potential to be an auxiliary biomarker of TNM stage to subdivide ESCC patients more accurately, which has important clinical significance.

## Introduction

Esophageal squamous cell carcinoma (ESCC) has always been a malignant gastrointestinal cancer tumor threatening human health worldwide, with high incidence and death rates (Torre et al., 2015; Chen et al., 2016). Despite the continuous development of therapeutic strategies including surgery, chemotherapy, and radiotherapy, the five-year survival rate of ESCC patients is still limited by 30–40% (Ferlay et al., 2015). A large amount of evidence indicated that tumor heterogeneity is one of the reasons for the poor clinical outcome of ESCC patients (Lin and Lin, 2019); therefore, patients exhibit distinct molecular profiles. Therefore, identification of molecular biomarkers is pivotal to predict the ESCC patients’ survival.

In recent decades, with the rapid development of computing platform of human transcriptome, microarray, and high-throughput sequencing technology, a large amount of omics data has been generated and stored in GEO and other large public databases, which will help us further reveal the molecular mechanism of tumorigenesis and explore tumor markers from the RNA level. Long non-coding RNA (lncRNA) is a type of RNA whose transcription length is >200 nucleotides and lacks the ability to encode proteins (Huarte, 2015). Accumulating evidence supports that lncRNAs can regulate both normal development and disease progression in various species (Mercer et al., 2009; Ulitsky and Bartel, 2013; Peng et al., 2017; Guo et al., 2019). Among them, a large number of lncRNAs have been regarded as critical molecules in promoting tumor growth and metastasis (Bhan et al., 2017), such as H19 (Ghafouri-Fard et al., 2020), MALAT1 (Hirata et al., 2015), PCAT-1 (Prensner et al., 2011), PCGEM1 (Srikantan et al., 2000; Shuo Chen et al., 2018), and HOTAIR (Gupta et al., 2010). In ESCC, lncRNAs, such as ZFAS1 (Li et al., 2019), CASC9 (Liang et al., 2018), GHET1 (Liu et al., 2017), TUSC7 (Chang et al., 2018), and FAM201A (Mingqiu Chen et al., 2018), have been suggested to involve in regulating ESCC epithelial–mesenchymal transition (EMT), metastasis, chemosensitivity, and radiosensitivity. Moreover, due to their high tissue- and cell-specific expression pattern, and their stability and detectability in body fluids, plasma, and urine, lncRNAs open up a new field for their applications as non-invasively diagnostic or prognostic biomarkers and therapeutic targets. A study by Feng et al. (2019) summarizes the observed lncRNAs that could be used as prognostic biomarkers of ESCC, such as SEMA3B-AS1, SNHG6, BANCR, UCA1 and MALAT1, FOXD2-AS1.

Gene expression profiling identifies many gene expression signatures from a variety of tumors, thereby enhancing our understanding of molecular alterations in the carcinogenic process and providing biomarkers for diagnosis or prognosis (Yang et al., 2020). In this research, we aim to find a prognostic biomarker for ESCC patients from the perspective of the lncRNA expression signature. Firstly, we downloaded both the lncRNA expression profiles and the matching clinical follow-up features from the GEO database. Then, Kaplan–Meier (KM) and Cox analyses were used to screen out the lncRNAs correlated with ESCC survival. Integrated bioinformatics methods were performed to establish a prognostic lncRNA signature and validate its prediction performance in multiple datasets. Finally, we confirmed that one of the lncRNAs LINC01273 may serve as an oncogene in ESCC.

## Materials and Methods

### Collection of ESCC RNA Expression Profiles

The ESCC RNA expression profiles and corresponding clinical information were obtained from the Gene Expression Omnibus (GEO, https://www.ncbi.nlm.nih.gov/geo/) database including GSE53624 (https://www.ncbi.nlm.nih.gov/geo/query/acc.cgi?acc=GSE53624), GSE53622 (https://www.ncbi.nlm.nih.gov/geo/query/acc.cgi?acc=GSE53622), and GSE53625 (https://www.ncbi.nlm.nih.gov/geo/query/acc.cgi?acc=GSE53625) datasets. Samples with complete survival information are retained, while those patients without survival information are eliminated. To develop prognostic prediction lncRNA models, ESCC samples from GSE53624 were treated as a training set. GSE53622 and GSE53625 sets were test and validation datasets. The aforementioned datasets were generated with Agilent-038314 (GPL18109). Through re-annotating microarray probes (see details in the Supplementary Material) (Harrow et al., 2012; White et al., 2014; Guo et al., 2018), we gained the expression values of lncRNAs from ESCC cohorts (Supplementary Table S1). Probes with missing expression values in more than 20% of patients were discarded.

### Construction of the Multi-lncRNA Predictive Models Related to Overall Survival

To single out those lncRNAs which were significantly associated with the prognosis of ESCC patients, both univariable Cox regression and KM survival analysis (the median lncRNA expression value as the cutoff value) were used in the training dataset. Those with Cox *p* < 0.05 and log rank *p* < 0.05 were considered OS-associated candidates. The LASSO regression method was then applied to obtain the strongest survival-related lncRNAs in the training set. Subsequently, the selected prognostic lncRNAs by KM, Cox, and LASSO regression were performed to develop combination models for estimating the ESCC prognosis risk as follows: risk score (RS) = ∑ Ni = 1 (Exp * coefficient), where N is the number of selected lncRNAs, Exp is the corresponding lncRNAs’ expression level, and the coefficient is calculated by the univariable Cox analysis. Based on the above formula, the RS of each combination model for each ESCC patient was calculated and ROC curve analysis was applied to make comparison of the survival prediction ability among those constructed multi-lncRNA signatures in the training set.

### Cell Culture and Cell Transfection

Human ESCC cell lines KYSE410 and TE5 were cultured in RPMI 1640 (Gibco) medium with 10% fetal bovine serum (TransSerum) and 1% streptomycin–penicillin solution (Gibco). All cells were cultured in a 5% CO_2_ constant temperature incubator. Small interfering RNAs (siRNAs) targeting LINC01273 (siLINC01273-1: 5′-GAC​ACA​GAA​GGA​CAA​UGU​UTT-3′; siLINC01273-2: 5′-GAC​ACA​AAG​UGA​CAG​AAU​GTT-3′) were synthesized by GenePharma Co. (Suzhou, China). Following the instructions, siLINC01273 was transfected at a concentration of 40 nM using Lipofectamine RNAiMAX Transfection Reagent (Invitrogen) with Opti-MEM (Gibco). After transfection for 48 h, the RNAs were harvested.

### RNA Extraction and RT-qPCR

Total RNA was reverse transcribed into cDNA by HiScript Q RT SuperMix for qPCR (Vazyme) after extracting by RNA-easy Isolation Reagent (Vazyme). The real-time RT-qPCR assay was conducted with an ABI 7500 system (Corbett Life Science) using ChamQ SYBR Color qPCR Master Mix (Vazyme) with the guide of its manufacturer’s instructions. The primers for RT-qPCR of LINC01273 were 5′-TGT​TGC​GGT​GTT​CAG​GGG​TTT-3′ (forward) and 5′-GTC​TGG​CTT​CTT​TCA​CTG​AGC-3′ (reverse). The primers for beta-actin were 5′-CAA​CTG​GGA​CGA​CAT​GGA​GAA​A-3′ (forward) and 5′-GAT​AGC​AAC​GTA​CAT​GGC​TGG​G-3′ (reverse). The relative mRNA expression was normalized to beta-actin as reference.

### Cell Proliferation Assays

For the MTS assay, after transfection for 36 h, 5,000 cells/well were seeded into 96-well plates. After adding MTS solution (Promega) and incubating for 2 h, the absorbance was recorded at 490 nm using an ELISA plate reader. For the colony formation assay, 500 cells/well were planted in 12-well plates and continuously grown for 2 weeks until a single colony was formed. After fixing with methanol, these colonies were stained with 0.1% crystal violet.

### Transwell

ESCC cells were transfected with siRNAs for 36 h, and then serum starvation was performed for 12 h. For invasion assays, upper transwell chambers (Falcon) should be pre-coated with Matrigel (BD Biosciences) and then left in the incubator for 1 h. 5×10^4^ cells in 200 μL serum-free cell suspensions were added in the upper transwell chambers, while 500 μL medium containing 10% FBS was added in the bottom chamber. 36 h later, pictures were taken with a microscope magnifying ×200 after fixing and then staining the migrated or invasive cells from upper chambers.

### Statistical and Bioinformatics Analysis

The 50th percentile of the risk score is defined as the threshold to classify the high-risk group and the low-risk group. KM analysis was applied to evaluate and validate the survival prediction performance of the lncRNA signature in different ESCC cohorts. The time-dependent ROC curve was used to compare the prediction ability of the lncRNA signature with that of other clinical features at different survival times. And univariable and multivariable Cox regression and stratification analysis were used to test whether the multi-lncRNA risk score model was independent of other clinical characters. The R program (3.5.1) including R packages named survival, survminer, glmnet, pROC, and timeROC was used to perform the above analyses.

To explore the potential biological functions of lncRNAs, the Pearson correlation test was used to construct co-expressed networks of lncRNAs and the protein-coding genes (PCGs) in the GSE53625 dataset, and the PCGs that were highly correlated with lncRNAs (correlation coefficient >0.60/< -0.6, *p* < 0.001) were selected for GO and KEGG pathway enrichment analysis by the Cluego plugin in Cytoscape (Guo et al., 2018). SubpathwayMiner was also used to identify related pathways of the co-expressed PCGs in the KEGG database including entire pathways and sub-pathways.

All experiments were repeated for at least three times. The values are shown as mean ± SD. Prism 8 software was used to perform statistical analyses. Student’s t-test was employed for comparisons between two groups, and one-way ANOVA was performed for multiple-group comparisons. The differences with *p < 0.05, **p < 0.01, ***p < 0.001 were considered statistically significant.

## Results

### ESCC Clinical Characteristics and Expression Profiles

There were a total of 179 ESCC samples used in this study, including 119 from GSE53624 and 60 from GSE53622, respectively. GSE53625 is the union of GSE53624 and GSE53622. The median survival age was 60 years. There were more male patients with ESCC than females (146 vs*.* 33), and most of the patients were dead (survival time, 3 days to 60 months). Other clinical characters are shown in Table 1. In addition, through re-annotating microarray probes, a total of 6,253 expressed lncRNAs and 17,434 expressed PCGs were obtained from GSE53624 and GSE53622.

**TABLE 1:** Clinical features of the ESCC patients from GEO.

Features	GSE53624	GSE53622	GSE53625
Age (years)			
≤60	61	29	90
>60	58	31	89
Sex			
Female	21	12	33
Male	98	48	146
Tumor grade			
G1	23	9	32
G2	64	34	98
G3	32	17	49
T stage			
1	8	4	12
2	20	7	27
3	62	48	110
4	29	1	30
N stage			
0	54	29	83
1	42	20	62
2	13	9	22
3	10	2	12
TNM stage			
1	6	4	10
2	47	30	77
3	66	26	92
Survival status			0
Alive	46	30	76
Dead	73	30	103

### Identification of the Prognostic lncRNAs in the Training Set

**FIGURE 1:**
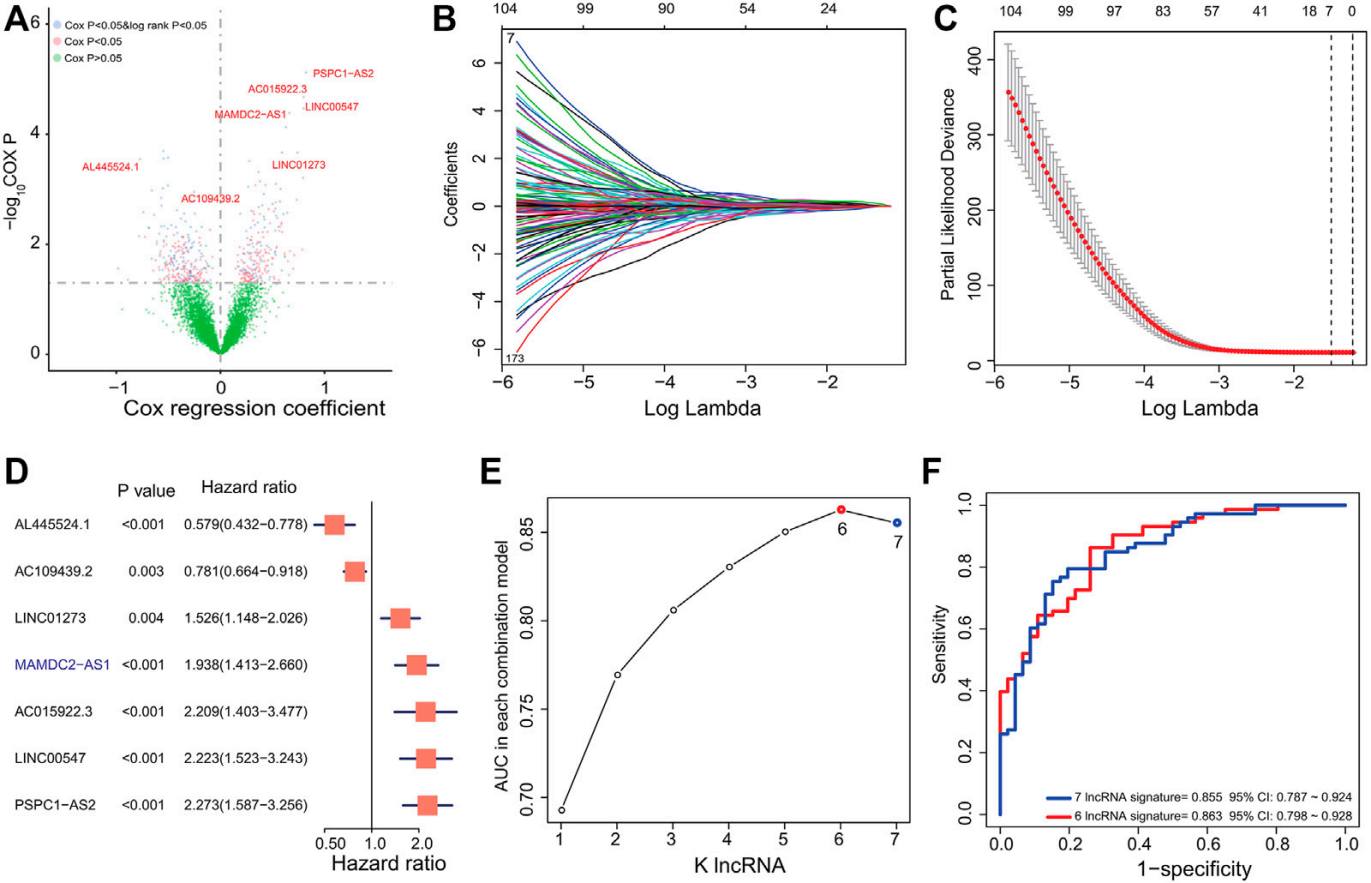
Derivation and selection of the lncRNA signature in the training dataset. **(A)** Univariate Cox regression and KM analysis identified 209 prognosis-related lncRNAs in the training dataset. **(B)** LASSO coefficient profiles for the 209-lncRNA set in the training dataset. **(C)** Cross-validation error rates for selecting the tuning parameters. **(D)** Hazard ratio of the selected lncRNAs by LASSO. **(E)** The AUC values of 127 multi-lncRNA signatures were calculated by ROC curve analysis. **(F)** ROC curve analysis for the 127 combinations and selected six-lncRNA signature in the training dataset.

ESCC samples from GSE53624 (*n* = 119) were treated as the training dataset to evaluate the relationship between ESCC OS and lncRNAs. After univariate Cox and KM analysis of lncRNAs’ expression level with clinical survival information, we identified a total of 209 lncRNAs (Figure 1A) related to ESCC patients’ OS significantly (Cox *p* < 0.05 and log rank *p* < 0.05), which could be used as prognostic candidates. Then, the LASSO regression algorithm via regression coefficient shrinkage based on a penalty that is proportional to size was utilized to screen out lncRNAs which were mostly correlated with ESCC survival among the 209-lncRNA set. As shown in Figure 1B, we found that the value of independent coefficients tended to zero with the increase of lambda value. Finally, we used threefold cross-validation and selected seven lncRNA candidates to construct the multi-lncRNA classifiers (Figure 1C).

### Construction of the Six-lncRNA Prognostic Signature

To select a better predictive multi-lncRNA model with fewer lncRNAs, ROC curve analysis was performed to compare the prognostic prediction performance of the 2^7^-1 = 127 risk score combinations in the training dataset (Supplementary Table S2). All risk scores for each ESCC based on the corresponding lncRNA signature were calculated as the method described. Then, the six-lncRNA combination with the largest AUC value composed by AL445524.1, AC109439.2, LINC01273, AC015922.3, LINC00547, and PSPC1-AS2 was obtained (Figure 1D; Table 2). The RS of the six-lncRNA signature is as follows: RS = (-0.5460037×AL445524.1) + (-0.2473264× AC109439.2) + (0.4223392× LINC01273) + (-0.81843 × AC015922.3) + (0.7987309× LINC00547) + (0.8210199× PSPC1-AS2). The AUC of the six-lncRNA signature was 0.863 (95% CI: 0.798–0.928), higher than that of the seven-lncRNA model (0.855, 95% CI: 0.787–0.924, Figures 1E,F) and other lncRNA combinations. Therefore, we chose the six-lncRNA signature with fewer nodes and better survival prediction ability as the candidate classifier.

**TABLE 2:** Prognostic significance of the six lncRNAs in the signature.

Ensembl ID	Gene name	HR	95% CI of HR		*p*	Chromosome location
			Lower	Upper		
ENSG00000233461	AL445524.1	0.579	0.432	0.778	<0.001	1:231520729-231528618: 1
ENSG00000250284	AC109439.2	0.781	0.664	0.918	0.003	5:136734830-136763409:1
ENSG00000231742	LINC01273	1.526	1.148	2.026	0.004	20:50171809-50176676:1
ENSG00000276855	AC015922.3	2.209	1.403	3.477	0.001	17:15789016-15789705:1
ENSG00000275226	LINC00547	2.223	1.523	3.243	<0.001	13:37534940-37551536:1
ENSG00000226352	PSPC1-AS2	2.273	1.587	3.256	<0.001	13:19674624-19675884:1

### Evaluation and Validation of the Prognostic lncRNA Model in ESCC

**FIGURE 2:**
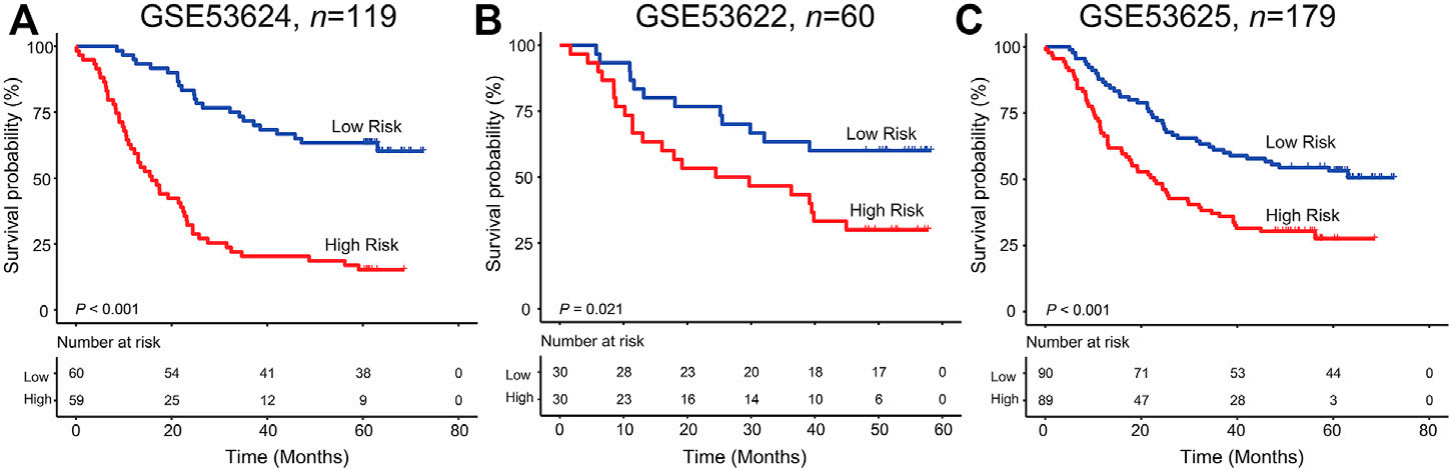
Kaplan–Meier analysis of the six-lncRNA signature in the GSE53624 **(A)**, GSE53622 **(B)**, and GSE53625**(C)** datasets.

In the GSE53624 set, on the basis of the median risk score calculated by the six-lncRNA signature, patients were distinguished into two groups with different OS. Unfortunately, patients with ESCC from the high-risk group suffered a worst survival outcome than those from the low-risk group (log rank *p <* 0.001, Figure 2A). The five-year survival rate of patients in the low-risk group was 63.3%, which was significantly more than 15.25% of patients in the high-risk group.

For verifying the survival classification power of the lncRNA model, each patient from the validation GSE53622 set obtained their risk score values. Figure 2B shows the KM curves for patients with ESCC from the low/high-risk group in the GSE53622 dataset. We found that the median survival time in the high-risk group was 39.17 months less than 50.6 months in the low-risk group (five-year survival rate: 30% *vs.* 60%, log rank test *p* = 0.021). As for the entire dataset (GSE53625), patients with high risk scores suffered more undesirable outcomes than those with low risk scores (median survival time: 23.13 months vs*.* 51.3 months; log rank test *p* < 0.001, Figure 2C).

**FIGURE 3:**
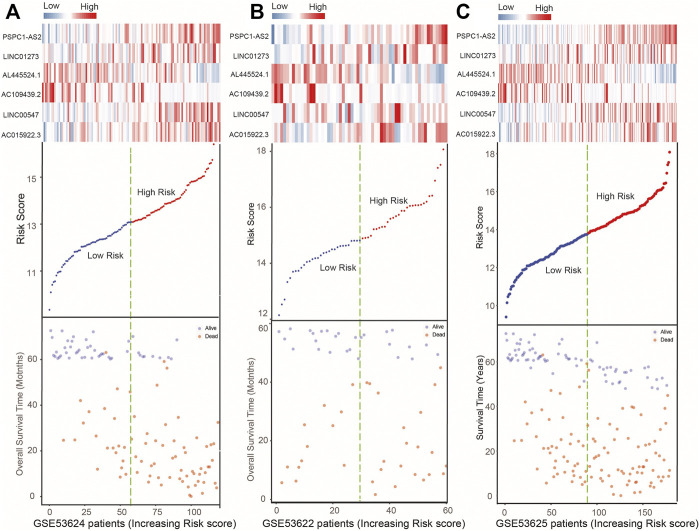
Expression heatmap of the six lncRNAs, plot of six-lncRNA risk scores, and ESCC patient’s survival status in the GSE53624 **(A)**, GSE53622 **(B)**, and GSE53625 **(C)** datasets.

Moreover, Figure 3 shows the lncRNAs’ expression pattern of ESCC patients, the distribution of survival status, and their risk scores. For ESCC patients with high risk scores from the training set, the expression values of four lncRNAs (LINC01273, AC015922.3, LINC00547, PSPC1-AS2) were high, while the expression values of protective lncRNAs (AL445524.1, AC109439.2) were low. In contrast, the expression of prognostic lncRNAs showed the opposite pattern in patients with low risk scores in the training set (Figure 3A). Subsequently, we confirmed the similar survival distribution and risky or protective lncRNAs’ expression pattern in GSE53622 and GSE53625 sets (Figures 3B,C).

### Evaluation of Survival Prediction Independence

To evaluate the independence of the signature in survival prediction with other clinical characters including age, gender, and TNM stage, Cox regression analysis in GSE53624, GSE53622, and GSE53625 datasets was performed, and the multivariate Cox results of the multiple ESCC datasets showed that the six-lncRNA signature in OS prediction was independent of age and gender (high vs*.* low risk, HR = 4.97, *p* < 0.001, *n* = 119; HR = 2.26, *p* = 0.025, *n* = 60; HR = 2.11, *p* < 0.001, *n* = 179, Table 3). In addition, TNM stage affected the OS of patients with ESCC in GSE53624, GSE53622, and GSE53625 datasets (III vs*.* I + II: HR = 1.8, *p* < 0.001, *n* = 119; HR = 2.37, *p* = 0.009, *n* = 60; HR = 1.95, *p* < 0.001, *n* = 179, Table 3).

**TABLE 3:** Cox regression analysis of the signature with ESCC survival.

		Univariable analysis				Multivariable analysis			
Variables		HR	95% CI of HR		*p*	HR	95% CI of HR		*p*
			Lower	Upper			Lower	Upper	
GSE53624									
Age	>60 *vs.* ≤60	1.42	0.90	2.25	0.14	1.66	1.02	2.72	0.04
Sex	Male *vs.* female	0.83	0.47	1.46	0.51	1.29	0.70	2.38	0.42
TNM stage	III *vs.* II, I	1.90	1.23	2.95	<0.001	1.80	1.15	2.83	0.01
Signature	High risk *vs.* low risk	4.50	2.71	7.46	<0.001	4.97	2.94	8.42	<0.001
GSE53622									
Age	>60 *vs.* ≤60	2.07	1.02	4.21	0.05	1.79	0.87	3.71	0.12
Sex	Male *vs.* female	0.71	0.31	1.64	0.42	0.54	0.22	1.34	0.18
TNM stage	III *vs.* II, I	2.12	1.15	3.91	0.02	2.37	1.24	4.53	0.01
Signature	High risk *vs.* low risk	2.26	1.11	4.61	0.02	2.26	1.11	4.60	0.03
GSE53625									
Age	>60 *vs.* ≤60	1.59	1.08	2.34	0.02	1.49	1.01	2.22	0.05
Sex	Male *vs.* female	0.78	0.49	1.25	0.31	0.80	0.49	1.30	0.37
TNM stage	III *vs.* II, I	1.99	1.40	2.85	<0.001	1.95	1.35	2.80	<0.001
Signature	High risk *vs.* low risk	2.12	1.43	3.14	<0.001	2.11	1.42	3.13	<0.001

### Comparison of the Six-lncRNA Signature With Clinical Features in Survival Prediction Ability

**FIGURE 4:**
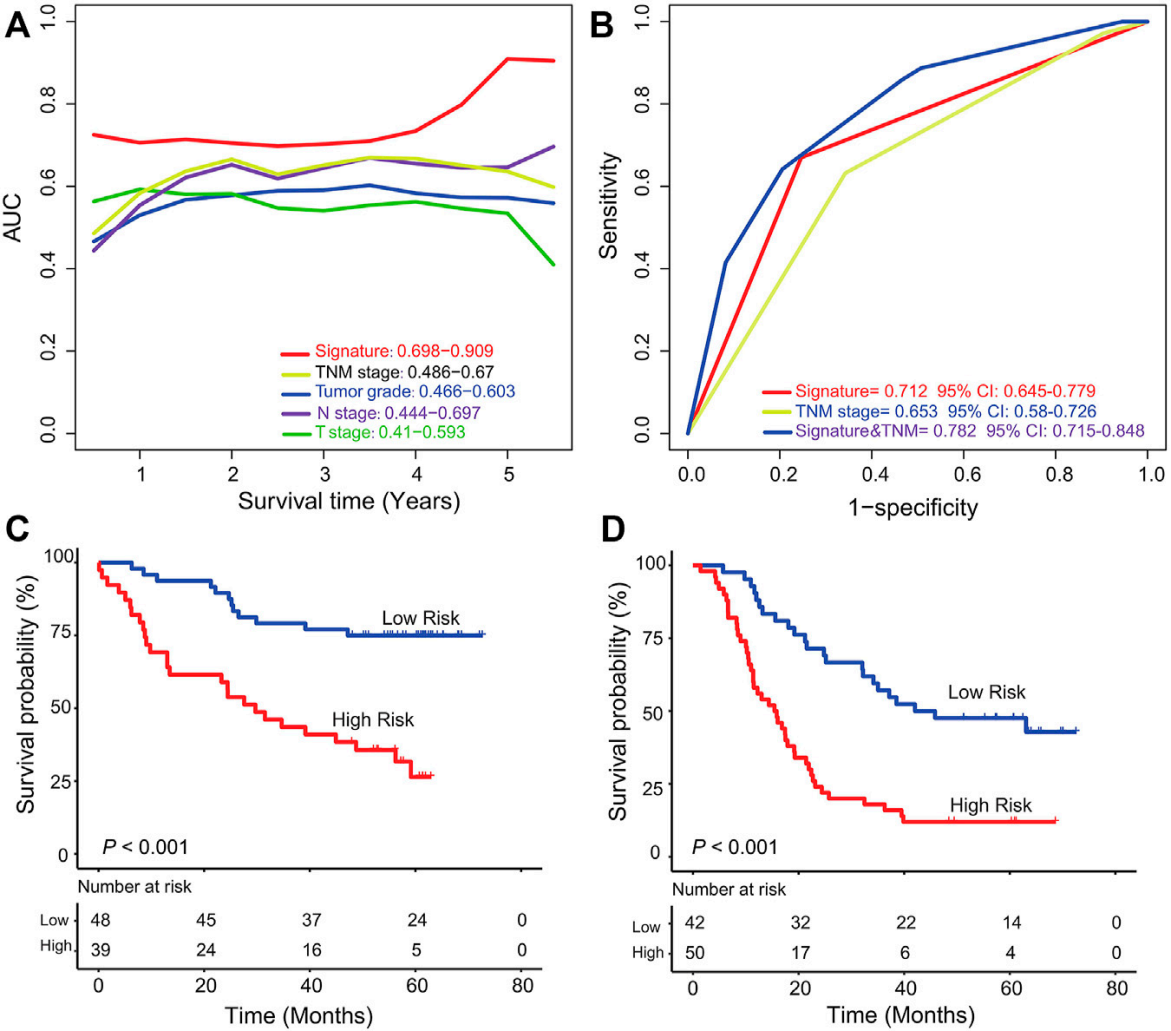
Comparison of TNM stage and the six-lncRNA signature and stratification analysis. **(A)** Time-dependent ROC curve analysis of the six-lncRNA signature and other clinical characters in the GSE53625 group. **(B)** Comparison of survival prediction performance of TNM stage and the six-lncRNA signature. The signature could further classify ESCC patients from TNM high **(C)**/low **(D)** stage into two groups according to markedly different survival.

Time-dependent ROC curve analysis from 1 year to 5 years was applied to compare the survival prediction ability of the lncRNA signature with that of tumor grade, TNM stage, T stage, and N stage in the entire ESCC group (GSE53625, *n* = 179). The AUC values showed the predictive ability of the lncRNA signature (AUC from 1 year to 5 years: 0.698–0.909) was better than that of TNM stage (AUC from 1 year to 5 years: 0.486–0.67) and other features, especially at 5 years (Figure 4A). And the AUC of the combined model was the largest one compared to that of TNM stage or signature alone (AUC = 0.712, 95% CI = 0.645–0.779, Figure 4B), which further suggested the signature has potential to become a novel prognostic biomarker.

### Stratification Analysis of the Six-lncRNA Signature

To evaluate whether the signature can further subgroup ESCC patients at high (III)/low (I, II) TNM stage, we performed stratification analysis in the entire dataset (GSE53625, *n* = 179). According to the TNM stage information of all the 179 patients, we found 87 patients at TNM low stage and 92 at TNM high stage. For patients at low TNM stage, the six-lncRNA signature could separate them into low- and high-risk groups with significantly different survival (five-year survival rate 59.1% vs*.* 18.6%, log rank test *p* < 0.001, Figure 4C). The signature can further classify patients at the high TNM stage into two groups with different prognostic outcomes (median survival: 28.7 months vs*.* 58.2 months; log rank test *p* < 0.001, Figure 4D). This result showed the potential ability of the six-lncRNA signature as a clinical auxiliary marker for TNM stage to subgroup patients with ESCC more accurately.

### Functional Prediction of lncRNAs From the Six-lncRNA Signature

The Pearson test observed that the expression of 491 PCGs was significantly related to at least one of the six prognostic lncRNAs (coefficient >0.60/< −0.6, *p* < 0.001). GO and KEGG function analysis was then performed by Cluego and SubpathwayMiner. The results showed the 491 PCGs correlated with lncRNAs were significantly enriched in 37 GO terms and 36 KEGG pathways (*p* < 0.05, Supplementary Table S3). All these vital GO terms were organized into an interaction network based on similar functions in Cytoscape, and several clusters of functionally related GO terms were found such as ncRNA metabolic process, RNA process via interacting with those PCGs that affect cell cycle, regulation of actin cytoskeleton, MAPK signaling pathway, cell cycle, and TGF−beta signaling pathway (Supplementary Figure S1B).

### Oncogenic Effect of LINC01273 in ESCC Cells

**FIGURE 5:**
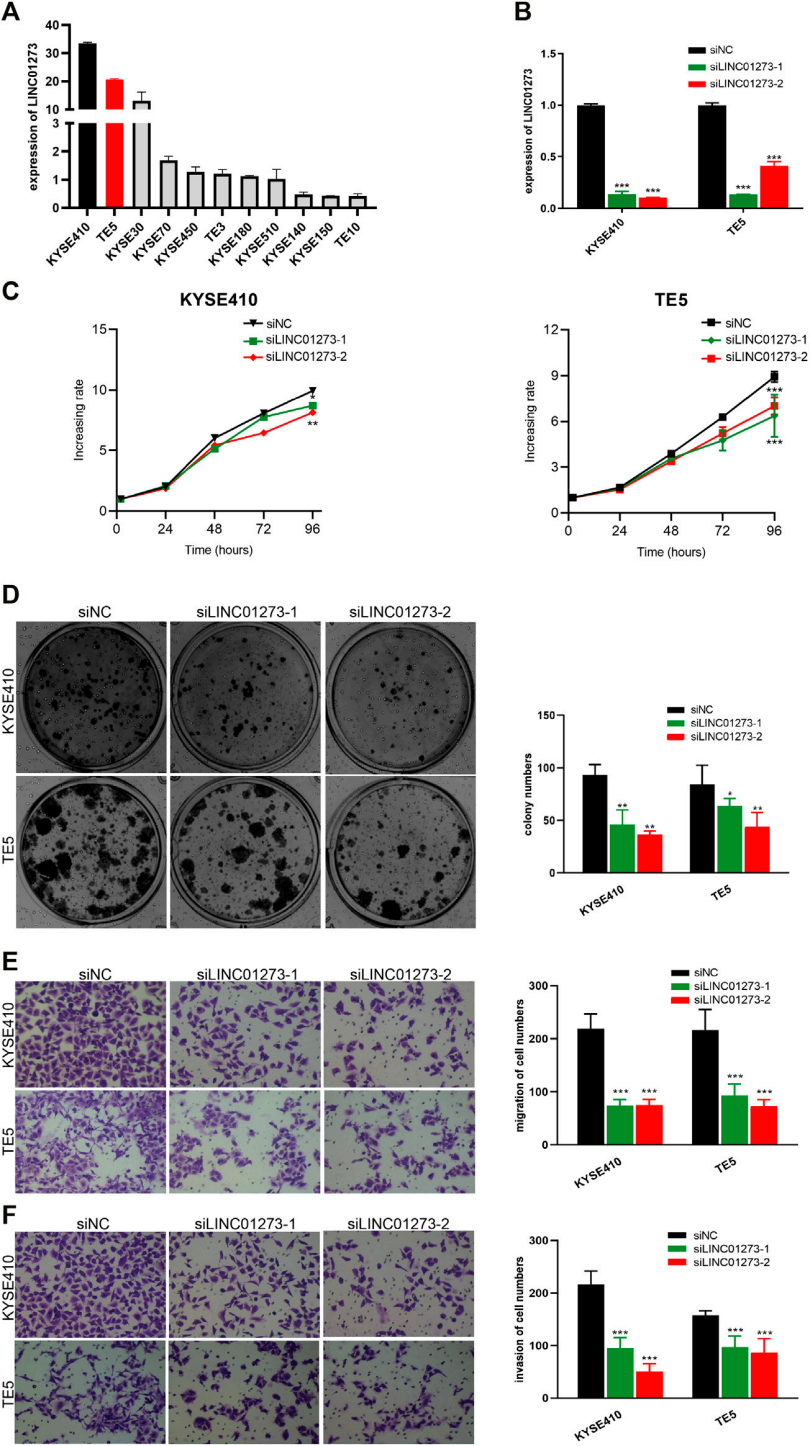
Oncogenic effect of LINC01273 on ESCC cells. **(A)** RT-qPCR analysis of LINC01273 expression in ESCC cell lines. **(B)** siRNA-mediated silencing of LINC01273 was evaluated by using RT-qPCR. **(C,D)** Results of the MTS assay **(C)** and colony formation assay **(D)** demonstrated that cell proliferation was inhibited after depletion of LINC01273 in KYSE410 and TE5 cells. **(E,F)** Transwell assays suggested that migration **(E)** and invasion **(F)** abilities were reduced after LINC01273 knockdown. All data are expressed as mean ± SD (*p < 0.05, **p < 0.01, ***p < 0.001).

We next investigated the biological roles of LINC01273 in maintaining the malignant phenotypes of ESCC cells. LINC01273 expression was examined in ESCC cell lines which our lab owned using qRT-PCR, and the results showed that LINC01273 was highly expressed in KYSE410 and TE5 cells (Figure 5A). Therefore, KYSE410 and TE5 cell lines were selected for further experiments. Firstly, we, respectively, transfected two individual siRNAs and confirmed LINC01273 was successfully knocked down by qRT-PCR (Figure 5B). We found that, by using the MTT assay and cell colony formation assay, silencing LINC01273 remarkably attenuated both the proliferation and colony formation capability of ESCC cells (Figures 5C,D). Transwell assays showed a significant suppression of the migration and invasive abilities of the two ESCC cell lines due to LINC01273 downregulation (Figures 5E,F). These results suggested that LINC01273 might enhance the ability of proliferation, migration, and invasion of KYSE410 and TE5 cells, demonstrating that LINC01273 may play oncogenic roles in ESCC.

## Discussion

Esophageal cancer ranks eighth in the global incidence of malignant tumors and sixth in tumor-related mortality. ESCC, the most common subtype of esophageal cancer, is so extremely aggressive that recent medical developments have not improved the prognosis of patients. TNM stage is still the main tool for predicting the survival of ESCC (Kang et al., 2020). However, ESCC patients with the same pathological characteristics at diagnosis often have completely different survival outcomes (Matsueda and Ishihara, 2020). For ESCC patients, the application of molecular characteristics to prognostic prediction may help resolve tumor heterogeneity and achieve precise treatment and evaluation. Accumulating evidence shows that lncRNAs are functional regulatory molecules in a variety of tumors. In ESCC, it is reported that lncRNAs regulate tumor progression through multiple mechanisms and multiple molecular interactions (Feng et al., 2019) and have the prognostic value because they are too closely related to survival (Deng et al., 2016). Therefore, exploring a prognostic lncRNA signature from ESCC patients would be meaningful and urgently necessary.

In this study, we achieved and re-mined the publicly available lncRNA profiles of ESCC (Li et al., 2014) and identified a total of 209 survival-related lncRNAs by KM and Cox survival analysis. Then, we developed a six-lncRNA model including AL445524.1, AC109439.2, LINC01273, AC015922.3, LINC00547, and PSPC1-AS2, which was significantly correlated with the prognosis of ESCC. Different from most of the existing prognostic model construction process (Zeng et al., 2018; Bao et al., 2019; Liu et al., 2019; Wang et al., 2019), following LASSO regression analysis which reduced the number of prognostic lncRNAs directly from 209 to 7, we added a key step, permutation and combination of the LASSO-selected lncRNAs, which further diminished the node number in the signature and greatly improved the clinical utility of the signature. Consistent with the risk model construction and prognostic signature screening methods reported in other literature (Guo et al., 2016), we further performed ROC curve analysis on RS models and screened the signature with the strongest predictive ability from multiple signatures composed of seven lncRNAs. In addition, because the AUC value of our six-lncRNA signature is greater than that of other signatures discovered by some researchers (Zhang et al., 2020), our signature performs better in prognostic prediction.

Moreover, we accessed the independence of the six-lncRNA signature from other ESCC clinical characters including age, sex, and TNM stage by Cox regression analysis in multiple ESCC datasets and showed it was an independent prognostic factor. ROC curve analysis results suggested the lncRNA signature had better accuracy in survival prediction than TNM stage, and the combination of TNM stage and lncRNA signature can evaluate the prognosis of patients more accurately. Stratified analysis indicated the ESCC patients at high/low TNM stages could be further separated into two different groups with significantly different survival. Taken together, the six-lncRNA signature could be a valuable classifier for ESCC prognosis and have potential to become an auxiliary biomarker for TNM stage to subdivide patients effectively.

As for the prognostic correlation of six prognostic lncRNAs, the high expression of four risk lncRNAs, LINC01273, AC015922.3, LINC00547, and PSPC1-AS2, was related to poor survival (Cox coefficient >0, *p* < 0.01), and the remaining protective lncRNAs (AL445524.1 and AC109439.2) were associated with longer survival time (Cox coefficient <0, *p* < 0.01). The biological functions of these six lncRNAs in cancer have not been reported until now. However, we have demonstrated that one of the lncRNAs of the six-lncRNA signature, LINC01273, may act as an oncogenic lncRNA to improve the abilities of proliferation, migration, and invasion in ESCC, which suggested the importance of LINC01273 in the six-lncRNA signature and other five lncRNAs may play key roles in ESCC as well. Moreover, our functional enrichment analysis results revealed that they may participate in tumorigenesis by cell cycles, MAPK signaling pathway, and TGF-beta signaling pathway. Accumulating studies suggested that the TGF-beta signaling pathway plays an important role in many kinds of cancers due to its importance in migration and EMT which is closely related to chemotherapy resistance (Colak and Ten Dijke, 2017).

So far, we have only demonstrated that LINC01273 may function as an oncogenic lncRNA. Although the potential function of these lncRNAs has been predicted by bioinformatics methods principally, the roles of these lncRNAs in ESCC are still unclear and need more experimental studies to further elucidate in the future. Another drawback of this study is that the model has not been tested and verified in clinical trials. Despite these shortcomings, the significant and consistent correlation between the lncRNA signature and OS in multiple ESCC datasets indicated that the six-lncRNA signature is a powerful prognostic marker of ESCC. Furthermore, our current experiment has confirmed the carcinogenic effect of LINC01273 on ESCC.

In conclusion, the six-lncRNA signature constructed in this study could predict the survival of ESCC patients more accurately and have the potential to be an auxiliary molecular biomarker of TNM stage in prognosis.

## Data Availability

Publicly available datasets were analyzed in this study. These data can be found here: ftp://ftp.ncbi.nlm.nih.gov/geo/series/GSE53nnn/GSE53624/matrix/ ftp://ftp.ncbi.nlm.nih.gov/geo/series/GSE53nnn/GSE53622/matrix/ ftp://ftp.ncbi.nlm.nih.gov/geo/series/GSE53nnn/GSE53625/matrix/.

## Supplementary Material

Supplementary Figure S1 Functional enrichment analysis of the six lncRNAs by Cluego **(A)** and SubpathwayMiner **(B)**.

Supplementary Table S1 Re-annotating probes for Agilent-038314 CBC *Homo sapiens* lncRNA + mRNA microarray V2.0.

Supplementary Table S2 127 signatures comprising different lncRNAs in the GSE53624 dataset (*n* =119).

Supplementary Table S3 Functional enrichment analysis of the six lncRNAs by Cluego and SubpathwayMiner.

## Abbreviations

AUC, area under the ROC curve; ESCC, esophageal squamous cell carcinoma; KM, Kaplan–Meier; OS, overall survival; ROC, receiver-operating characteristic.
