# Supplementary figures and images for: Construction of the Six-lncRNA Prognosis Signature as a Novel Biomarker in Esophageal Squamous Cell Carcinoma
Source: Front Genet. 2022 Mar 31;13:839589. doi: 10.3389/fgene.2022.839589 (PMC9008717; doi:10.3389/fgene.2022.839589)

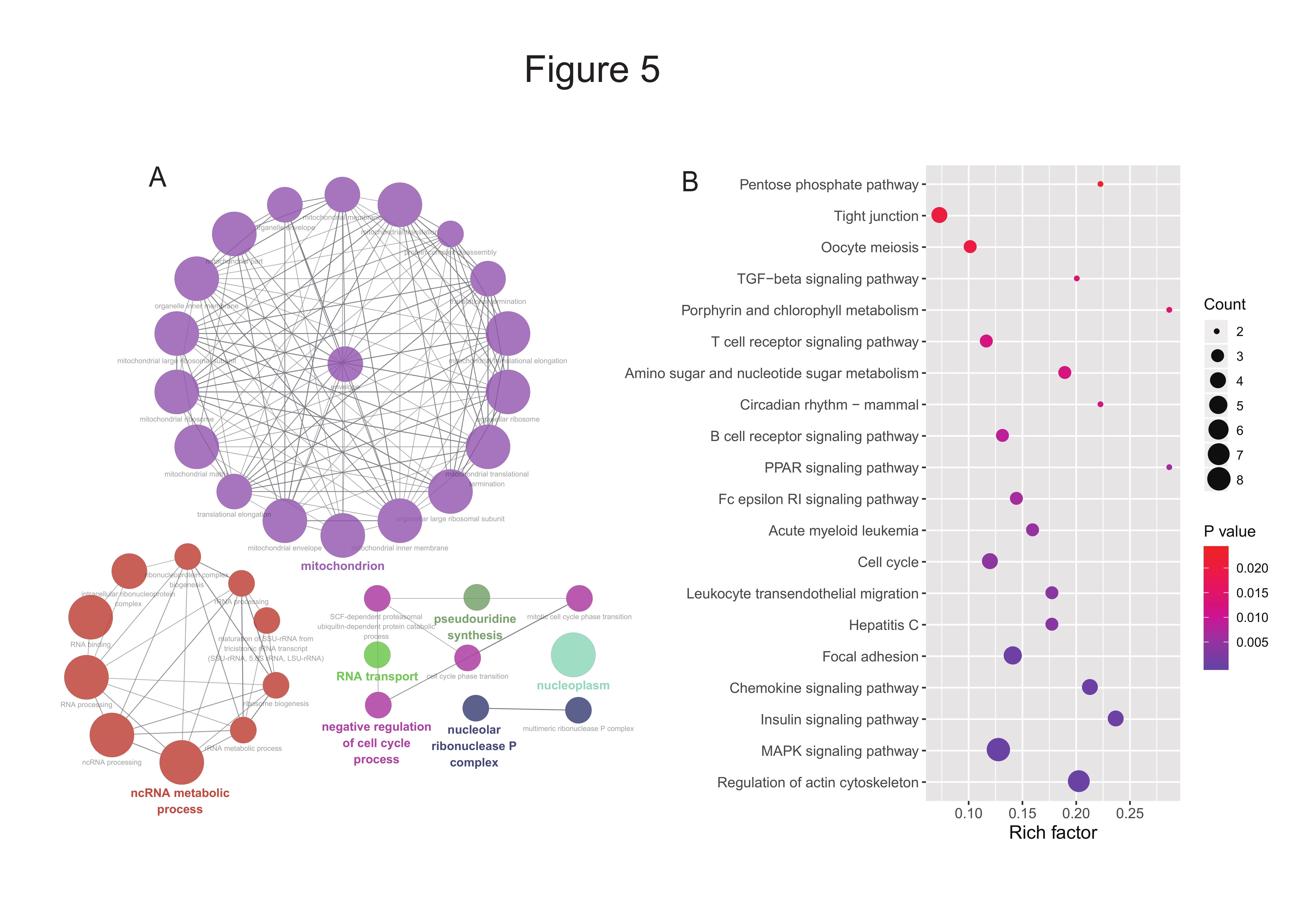

Supplement: Supplementary file 3 [file Image1.JPEG]
